# Nano-Fertilization as an Emerging Fertilization Technique: Why Can Modern Agriculture Benefit from Its Use?

**DOI:** 10.3390/plants10010002

**Published:** 2020-12-22

**Authors:** Mahmoud F. Seleiman, Khalid F. Almutairi, Majed Alotaibi, Ashwag Shami, Bushra Ahmed Alhammad, Martin Leonardo Battaglia

**Affiliations:** 1 Plant Production Department, College of Food and Agriculture Sciences, King Saud University, P.O. Box 2460, Riyadh 11451, Saudi Arabia; almutairik@ksu.edu.sa (K.F.A.); malotaibia@ksu.edu.sa (M.A.); 2 Department of Crop Sciences, Faculty of Agriculture, Menoufia University, Shibin El-kom 32514, Egypt; 3 Biology Department, College of Sciences, Princess Nourah bint Abdulrahman University, Riyadh 11617, Saudi Arabia; ayshami@pnu.edu.sa; 4 Biology Department, College of Science and Humanity Studies, Prince Sattam Bin Abdulaziz University, Al Kharj Box 292, Riyadh 11942, Saudi Arabia; b.alhamad@psau.edu.sa; 5 Department of Animal Sciences, Cornell University, Ithaca, NY 14850, USA

**Keywords:** NFs, NPs, sustainable agriculture, crop production, plant nutrition, biotic and abiotic stress

## Abstract

There is a need for a more innovative fertilizer approach that can increase the productivity of agricultural systems and be more environmentally friendly than synthetic fertilizers. In this article, we reviewed the recent development and potential benefits derived from the use of nanofertilizers (NFs) in modern agriculture. NFs have the potential to promote sustainable agriculture and increase overall crop productivity, mainly by increasing the nutrient use efficiency (NUE) of field and greenhouse crops. NFs can release their nutrients at a slow and steady pace, either when applied alone or in combination with synthetic or organic fertilizers. They can release their nutrients in 40–50 days, while synthetic fertilizers do the same in 4–10 days. Moreover, NFs can increase the tolerance of plants against biotic and abiotic stresses. Here, the advantages of NFs over synthetic fertilizers, as well as the different types of macro and micro NFs, are discussed in detail. Furthermore, the application of NFs in smart sustainable agriculture and the role of NFs in the mitigation of biotic and abiotic stress on plants is presented. Though NF applications may have many benefits for sustainable agriculture, there are some concerns related to the release of nanoparticles (NPs) from NFs into the environment, with the subsequent detrimental effects that this could have on both human and animal health. Future research should explore green synthesized and biosynthesized NFs, their safe use, bioavailability, and toxicity concerns.

## 1. Introduction

**Figure 1:**
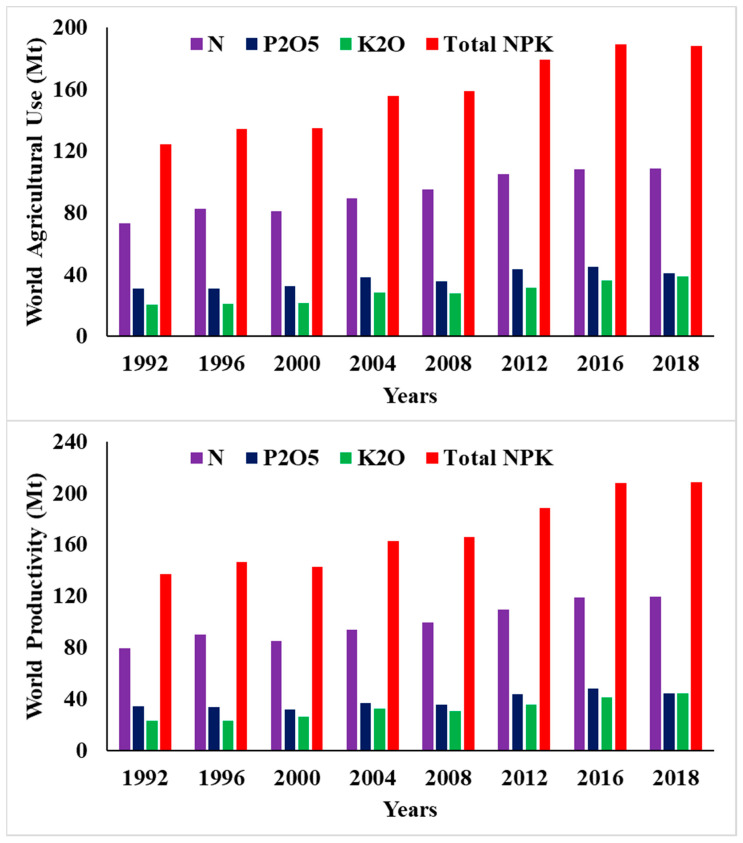
Total agricultural use and productivity of nitrogen (N), phosphorus (P) and potassium (K) fertilizers worldwide [4].

Sustainable agriculture with a high productivity is crucial to alleviate the perils of hunger and increase food security. Food production and distribution are under an increased and continuous stress at a global scale due to climate change, an increased human population, and decreased fertile lands and freshwater resources [1]. This challenge could be addressed with technological advancements coupled with significant modifications to existing global food production systems [2,3]. Currently, modern agriculture is heavily supported by the use of high rates of agrochemicals. For example, the global production of synthetic fertilizers amounted to 188.2 Mt in 2019 (Figure 1) [4], while the current application of pesticides is about 4 Mt in agricultural fields [5]. It is expected that this amount of agrochemicals will be increased in the near future to an amount that could feed 9.6 billion people by 2050 [6,7,8]. Synthetic chemical fertilizers are used for the optimal growth and productivity of crops, but, at present, adopted agricultural practices have not been particularly successful to simultaneously enhance plant nutrient uptake, nutrient use efficiency (NUE), and crop productivity [8,9]. In most cases, synthetic fertilizers used in extensive agriculture have low NUE values [10]. For example, the NUE values of the three most basic macronutrients, i.e., nitrogen (N), phosphorus (P), and potassium (K), are low at 30–35%, 18–20%, and 35–40%, respectively [11,12], which shows that more than half of the broadcasted fertilizers in the fields are lost and do not reach their targeted sites due to different factors such as photolysis, hydrolysis, leaching, and microbial immobilization and degradation [13]. 

A low NUE can led to the intensive use of synthetic fertilizers to increase crop production [10]. However, in the long term, this intensive application of synthetic fertilizers can result in severe environmental risks such as air pollution, soil degradation, water eutrophication, and groundwater pollution [8,14,15]. Furthermore, the over-application of synthetic fertilizers increases the cost of their production and decreases the profit margin of farmers [7,8]. Low NUE values [16] and increased environmental risks [15] related to the use of more synthetic fertilizers has been a long-term limitation to achieve sustainability in agriculture [7,17]. Higher release levels of synthetic fertilizers than plant uptake levels or changes of the forms of nutrients into those which are not bioavailable to plants are typically the main result of low NUE values.

Therefore, sustainability in agriculture can be achieved through the implementation and utilization of innovative techniques [3] that could enhance global food production while also protecting natural and environmental resources [18]. Recent studies have suggested that nanotechnology may have a potential for modifying the current synthetic framework utilized in modern agriculture systems [19] by increasing the efficiency of novel agrochemicals [20] and providing solutions for environmental and agricultural problems [1]. Thus, research regarding the use of nanoparticles (NPs) has gained attention among agricultural researchers in recent years [5,20,21]. In this context, there is a scientific curiosity for the production of novel sources of fertilizers with the aim of increasing fertilizer use efficiency [22]. From a sustainable agriculture perspective, nanotechnology has the potential to develop new innovative types of fertilizers such as nanofertilizers (NFs) to increase global food production to feed the increasing world population [7,21,23].

**Figure 2:**
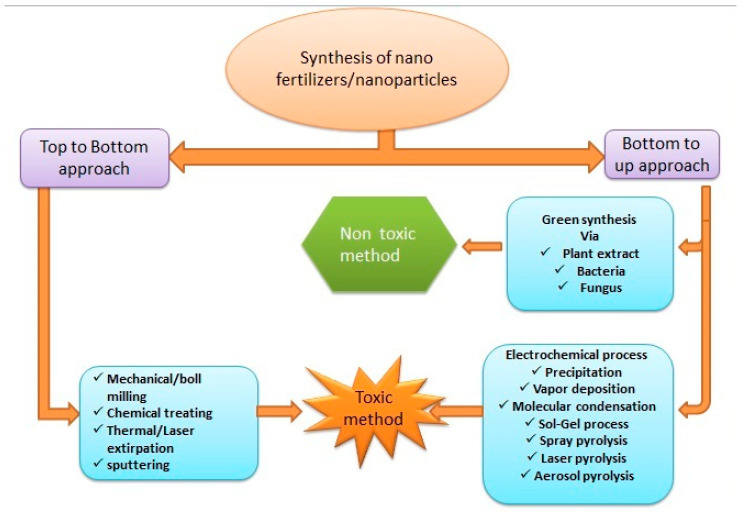
Main approaches for the synthesis of nanofertilizers (NFs) or/and nanoparticles (NPs).

The term nanofertilizer indicates that the nanomaterial, which is either a plant nutrient itself (micro- or macro-nutrients) or the carrier of a plant nutrient, is termed a nanofertilizer. Nutrients encapsulated or covered by nanomaterials are also called NFs [24]. NFs can be developed from synthetic substances (i.e., modified forms of synthetic fertilizers) or green synthesized from different parts of plants through various chemical, mechanical, or biological methods using nanotechnology [25] (Figure 2). There are two main approaches for the synthesis of nanoparticles: (i) the top to down and (ii) bottom to up approaches (Figure 2). In the top to down approach, nanoparticles are prepared by breaking down a bulk into nano-sized particles. In the bottom to up approach, nanoparticles are synthesized from atoms, molecules, and smaller monomers. Green synthesis is a non-toxic and environmental friendly method because it uses bio-organisms such as plants, fungi, and bacteria for the synthesis of nanoparticles, these micro-organisms work as both reducing and stabilizing agents, and no harmful substances or chemicals are used [26]. On the other hand, a chemical method that can be a top to down or bottom to up approach uses metal precursors, reducing agents, and toxic chemicals for the stability of nanomaterials that lead to harmful effects on humans and the environment when disposed [27]. NFs are used to increase soil fertility, the bioavailability of plant nutrients [16,25], and product quality [28]. Based on the nutrient requirements of plants, NFs are usually categorized into macro NFs, micro NFs, and nanoparticulate fertilizers [16]. NFs have large surface areas and a characteristic slow and steady release of nutrients, both of which make them highly suitable for use in modern agriculture [19,21,29].

Among other factors, crop productivity depends on the use of fertilizers. However, the high application rates of synthetic fertilizers have exhausted agricultural soils, decreased fertility, and decreased overall crop productivity [30,31,32,33,34]. According to some recent estimations, the use of fertilizers might account for about 30% of attained crop productivity, while the remaining 70% would depend on the efficient use of other factors and agricultural inputs [35]. However, a large portion of applied fertilizers are fixed within the soil or lost to the environment through volatilization, leaching, and water runoff [14], which results in a drastic reduction of the use efficiency of synthetic fertilizers [11]. For instance, the NUE values of N, P, and K are about 35%, 20%, and 40%, respectively [11,12]. Therefore, small quantities of fertilizers actually reach the targeted sites in plants, which results in the sub-optimum availability of nutrients at the plant level [36]. Consequently, farmers use higher than optimum doses of synthetic fertilizers to obtain high yields, which increases the concentration of salts and affects the inherent nutrient equilibrium of soils, ultimately negatively affecting plant productivity. Reports have shown that to produce 1.0 mg of grain, approximately 27 kg of NPK ha^−1^ was needed during the early 1970s, while 109 kg of NPK ha^−1^ was needed to achieve the same productivity in 2008 [37]. This depends on the climate zones and the types of soil and plants. Thus, it is of paramount importance to develop innovative fertilizers that can release their nutrients in a slow and steady way in order to increase crop yield, improve quality, and improve the overall sustainability of agricultural systems.

## 2. Advantages of NFs over Synthetic Fertilizers and Their Use for Sustainable Agriculture

NFs are coated or encapsulated with a nanomaterial that controls the release of nutrients according to the plant requirements, and this results in an increase in the NUE values of plants [38]. Remarkably, NFs can release their nutrients in 40–50 days, while synthetic fertilizers do the same in 4–10 days. As a result, a synthetic urea fertilizer can rapidly lose more than 70% of its N content after field application through leaching and volatilization, leaving less than 20% to be readily available for plants [39]. Recently, N was prepared in the NF form by coating the urea with hydroxyapatite NPs, which led a slow release of N to plants [40]. Similarly, research has shown that a nanohybrid of urea (i.e., modified form of hydroxyapatite) can release N as much as 12 times slower than synthetic urea in rice (*Oryza sativa* L.) fields, and it can increase grain yields at only 50% the rate used with common urea [40].

Similarly, synthetic P fertilizers have low uptake efficiencies and high fixation rates in the soil [41], while nano-formulations of P can reduce nutrient losses via the direct internalization of crops [2]. For example, the use of porous nanomaterials, such as chitosan and zeolites, has been found to considerably improve uptake efficiency by controlling demand-based release and decreasing the loss of N [42,43]. This depends on the method of NF application, whether foliar or soil amendments as explained in the following sections, since this can lead to different mechanisms of NF uptake and translocation in plants. Moreover, though numerous techniques have been recently proposed to increase P uptake efficiency (see review [33]), the success of such techniques has been limited. However, the high solubility of phosphate minerals and increased P uptake by plants was recorded after the application of ammonium zeolites [2]. On the other hand, the use of P-enriched hydroxyapatite NPs was found to considerably increase the plant height, shoot growth, and grain yield (18%) of soybeans (*Glycine max* L.) compared to plants grown with synthetic P fertilizers [44]. Likewise, carbon-based nanomaterials (e.g., graphene oxide films) have the potential to extend the process of potassium nitrate release, thus decreasing leaching losses [45]. 

Nanoparticles also influence some plant metabolic processes that influence the potential to mobilize nutrients like P in plants [46]. For example, to increase the uptake efficiency of synthetic P, zinc NPs are used for mobilization (see review [33]). Furthermore, biosensors can be attached to NFs to control the release of the nutrients and their bioavailability depending on the growth stage of the crop [47], a technology that is not applicable to synthetic fertilizers. Finally, the rates and costs of NF applications are typically lower than synthetic fertilizers, since NFs are required in small quantities [47]. 

## 3. Important Characteristics of NFs for Facilitating High Nutrient Use Efficiency (NUE) and Reducing the Leaching of Nutrients

**Figure 3:**
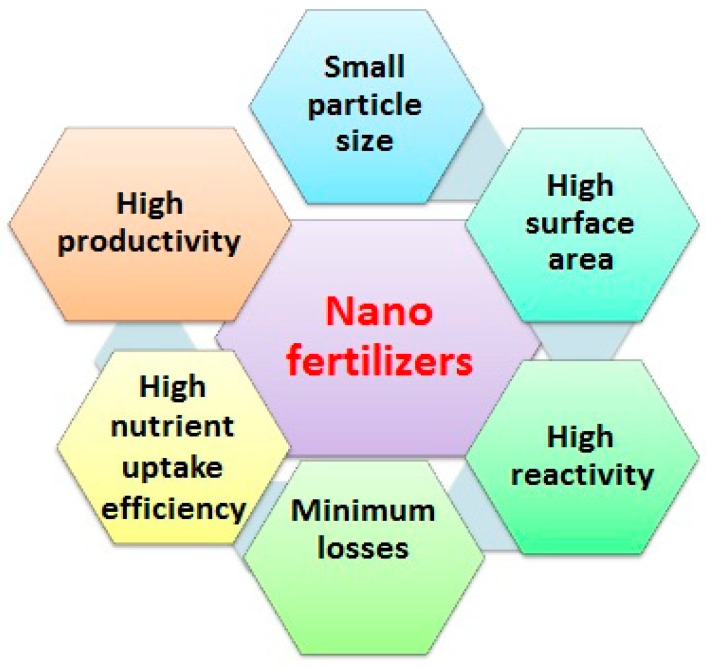
The most important advantages of NFs or/and NPs.

NFs have unique characteristics (Figure 3) that make them more beneficial than synthetic fertilizers [25,29]. One of the most important features of NFs is their ability to enter plants when applied as foliar or soil amendments due to their small particle size (<100 nm) [48]. NFs have high surface areas, and this can provide a maximum reactivity and increase both the availability of nutrients and plant NUE (Figure 2) [48,49]. Moreover, NFs are soluble in water and can increase the dispersion of nutrients in soil and further increase their availability to plants, while synthetic fertilizers have a low solubility due to their large particle size and high adsorption and fixation to soil particles. Fertilizers are encapsulated in NPs to increase their uptake and availability to plants, as well as to decrease their bulk requirements [16]. For example, the high availability of nutrients to plants throughout the growth period is possible via the application of zeolite-based NFs. Furthermore, the slow and targeted nutrient release [25] of NFs [49] minimizes their toxicity to plants [50] and decreases N losses via volatilization, leaching, fixation, and denitrification, as well as salt accumulation in soil.

## 4. Mechanisms of NF Uptake by Plants

It is very important to investigate the uptake and movement of NFs from soil into plants, because such information can give an idea of suitable applications for NFs to plants. For instance, if NFs or NPs prefer transport through the xylem, then the optimal application of NFs is through an irrigation system. Meanwhile, if NFs move through the phloem, then an exogenous application is recommended and suitable [29]. The composition of NFs, the size of NPs, the physiology of plants, and the pore diameter (5–20 nm) of the cell wall [29,51] affect the transportation and accumulation of nutrients released from NFs in plants [52]. 

### 4.1. Foliar Exposure and Uptake of NFs/NPs

In foliar applications, NPs face the cuticular barrier before entering plant tissues [53]. The cuticle layer is a waxy coating on leaves that has two entry points, i.e., the lipophilic or cuticular pathway and the hydrophilic or stomatal pathway. The lipophilic pathway is for nonpolar solutes that can enter the leaves through diffusion, while the hydrophilic pathway is for polar solutes [54]. Accordingly, NPs or their aggregates that are smaller than 4.8 nm in diameter can easily directly enter the cuticle through the cuticular pathway. However, several studies have documented that NPs larger than 5.0 nm can enter plants by foliar application (see review [55]). Polar nanoparticles, on the other hand, can enter though the hydrophilic or stomatal pathway [54]. However, differences in leaf morphology and the number and size of stomata among plant species can affect the uptake of foliar NPs [56]. The morphological dimensions of stomata are about 25 μm of length and 3–10 μm of width [54]. Nevertheless, as a result of the physiological function and unique geometric structure of stomata, the real size exclusion limit of a stomatal hole for NP diffusion is still unclear.

Following the stomatal pathway, nanoparticles can move long distances through a plant’s vascular system after entering the leaf apoplast (see review [55]). Since the vascular systems of plants are unidirectional and noncirculatory, the nutrients or photosynthates moving towards shoots (xylem) or roots (phloem) do not come back to their original sites [57]. Therefore, foliar-applied nanoparticles only have the phloem system option for uptake and translocation from leaves to roots. Wang et al. [58] observed that foliar-applied micronutrient oxide NPs (24–47 nm in diameter) on watermelon (*Citrullus lanatus* Thunb.) penetrated the leaves by following the stomatal pathway and reached the watermelon roots through sieve tube phloem. However, after the foliar spray of Cu NPs onto lettuce (*Lactuca sativa* L.), Zhao et al. [59] observed that 97–99% of Cu NPs were confined to the leaves of the lettuce, while only 1–3% were found in the root tissues. Wang et al. [60] documented that soil-applied CuO NPs (20–40 nm) translocated from the roots of maize (*Zea mays* L.) to the shoots through the xylem, and then they moved back to the roots through the phloem. Thus, the findings of Wang et al. [60] supported the concept that NPs can circulate within a plant both through the xylem and the phloem.

### 4.2. Root Exposure and Uptake of NFs/NPs in Plants

Multiple factors such as plant morphology, growth stage, exposure conditions, size of particles, and rhizosphere processes affect the root uptake of NPs. Slomberg and Schoenfisch [61] found that silicon (SiO_2_) nanoparticles with diameters between 50 and 200 nm entered the roots of *Arabidopsis thaliana*. Conversely, TiO_2_ NPs between 36 and 140 nm in diameter remained in the root parenchyma of wheat (*Triticum aestivum* L.) without reaching the vascular system, while NPs with a diameter of <36 nm were stored in the roots of the wheat and then translocated to the rest of the plant [62].

Surface charge also influences the uptake and translocation of NPs in plants (see review [55]). Avellan et al. [63] observed that the roots of *Arabidopsis thaliana* produced a mucilage that facilitated the taking up of positively-charged gold NPs (12 nm) by the roots, while same size (12 nm) of negatively charged gold NPs did not enter the root tissues. On the other hand, different plant species have shown different uptake capabilities for NPs, probably because of the variations in physiological and metabolic functions (see review [55]). For example, the roots of wild *Azolla caroliniana* absorbed both 4 and 18 nm gold NPs, and *Myriophyllum simulans* Orch absorbed only 4 nm gold NPs, while *Egeria densa* Planch did not uptake gold NPs of any diameter [64]. Likewise, Judy et al. [65] noted that the roots of tobacco plants uptake gold NPs ranging from 10 to 50 nm in diameter, whereas wheat roots do not. 

Nanoparticles applied to soil are initially adsorbed on the surface of roots and then cross several barriers to reach a plant’s vascular system (see review [55]). The first barrier is the root cuticle layer, which has a similar composition to that of the leaf cuticle layer. Nanoparticles cross the root surface cuticle and reach the root epidermis. When NPs reach the root epidermis, they could either follow the apoplastic or symplastic pathways. Many studies have documented that in the apoplastic pathway, NPs firstly enter the cell wall pores and then move into intercellular spaces (see review [55]). However, the pore diameter of the cell wall, typically ranging between 5 and 20 nm, restricts the passage of NPs through the apoplastic pathway to particles that have a diameter of less than 20 nm. Despite this, it might be possible for NPs to induce the destruction of the cell, and this would extend the size of pores. Additionally, it has been demonstrated that NPs can reach intercellular space in roots that have been exposed to diseases and herbivory by insects and soil microorganisms. Nevertheless, the main barrier in the apoplastic pathway is the Casparian strip around the vascular system, which prevents the direct entry of NPs to the vascular cylinder [66], although research has shown that ZnO NPs (30 nm) were able to enter from the lateral root junction of maize to then reach the vascular system [67]. Another possible route is the symplastic pathway, where NPs move from one cell to another through plasmodesmata [51,55]. When nanoparticles reach the central cylinder, they can then move to the aboveground parts of the plant via transpiration stream through the xylem [62].

In conclusion, NFs or/and NPs can be taken up by roots when they are applied to the soil or by leaves when they are foliar-applied. The exogenous application of NPs or their aggregates with smaller diameters can allow them to more easily enter the cuticle through the cuticular pathway than bigger NPs; however, differences in leaf morphology and in the number and size of stomata among plant species can affect the uptake of different NPs with different diameters. In soil application, NF or/and NP translocation can follow the apoplastic or symplastic pathways. In the apoplastic pathway, NPs firstly enter the cell wall pores and then translocate into intercellular space. Nevertheless, the pore diameter of the cell wall restricts NPs that are bigger than 20 nm through the apoplastic pathway. It is clear that the diameters of NFs or/and NPs have to be smaller in foliar application than in soil application.

## 5. Macronutrient (NPK) NFs and Their Effects on Plants

Fertilizers are indispensable for growth and for the enhancing yield and quality of crops, since they can provide the required nutrients for plant development. Macronutrients such N, P, and K are needed by plants in large quantities [15,30,32,68]. Since most of these nutrients are not efficiently taken up by plants, farmers tend to use high fertilizer doses to partially remediate their low NUE values, which results in a notoriously detrimental impact on soil, water, and the overall environment [8,15,16,69]. The use of NFs can increase the NUE of fertilizers, enhance crop yield and quality, and decrease the negative effects of synthetic fertilizers in the context of more sustainable agriculture [21,44,68]. NFs or nano-enabled fertilizers precisely release nutrients in the root zone of plants by preventing rapid changes in the chemical composition of the nutrients in the soil, which, in turn, reduces nutrient losses. Different types of NFs are produced depending on the material or the carrier present in them, e.g., hydroxyapatite nanoparticles, zeolite, mesoporous silica nanoparticles, nitrogen, copper, zinc, silica, carbon, and polymeric nanoparticles [10,48,70]. In Table 1, we present the type and the range doses of NF types based on different investigations and plant types, as well as their effects on plant growth and their philological, biochemical, and productivity traits.

### 5.1. Nitrogen NFs 

Nitrogen (N), considered the most important mineral nutrient for plants, is a basic part of several amino acids, proteins, DNA (deoxyribonucleic acid), ATP (adenine triphosphate), chlorophylls, and structural units of cells. Most of the metabolic functions and regulatory pathways in plants depend on adequate amounts of N. Plants uptake N in the forms of NO^−3^ and NH^+4^ [8,17]. One of the main constraints of synthetic N fertilizers is the high volatilization and leaching rates that occur during and immediately after their field application. To minimize these losses, N-based NFs could be utilized for the continuous supply of N at a slow release rate. Manikandan and Subramanian [97] used a zeo-urea nanofertilizer (N-NF) on maize plants and reported a high nutrient uptake, vigorous plant growth and yield, and better grain quality compared to synthetic urea fertilizers. Likewise, Mahmoodi et al. [98] applied N-NF to starflower (*Borago officinalis* L.) and reported a significant improvement in plant growth, which subsequently resulted in higher essential oil yields. Similarly, urea-modified zeolites were found to increase the seed yield of soybean (*Glycine max* L.) over synthetic fertilizers [44]. An N-NF developed by coating urea onto nanofilm was successfully used in *Brassica napus* L. [24]. Similarly, both nano-N and chelated nano-N were effective in terms of increasing the yield of a potato crop (*Solanum tuberosum* L.) and decreasing nitrate leaching [99]. Recently, Ha et al. [100] used NPK-coated NFs on coffee seedlings grown under greenhouse conditions. The authors documented that such NPK NF application increased the nutrient uptake and growth of coffee plants through an increase in the number of leaves and photosynthetic plant area. Moreover, the authors reported that NPK contents in plants increased up to 17.1%, 16.3%, and 67.5%, while the total chlorophyll and net photosynthesis rate increased up to 30.7% and 71.7%, respectively, compared to a control (zero NF). In conclusion, nitrogen in the form of NFs is highly recommended because it can cause a slow release of N, reduce volatilization and leaching rates, lead to a high nutrient uptake, and improve the growth and productivity of crops.

### 5.2. Phosphorus NFs 

After N, phosphorus (P) is considered to be the second most vital nutrient for optimum plant growth, as it is an integral part of energy transfer molecules, ATP, ADP (adenine triphosphate), phospholipids, and sugar phosphate, and it has a vital role in processes such as photosynthesis, respiration, and the biosynthesis of DNA [101]. Different parameters of plant productivity such as root and shoot length, plant vigor, resistance to diseases, number of reproductive buds, yield, and quality are strongly influenced by the availability of P [17]. However, P in synthetic fertilizers is poorly available due to its slow releasing time and high fixation in soils. A recent study showed that NFs can gradually deliver P for up to 40–50 days following their application, while common P synthetic fertilizers deliver all the nutrients within 8–10 days post-application [44]. Therefore, it has been suggested that the use of NFs or slow release materials like zeolites may have the potential to increase the NUE of P for several field crops [44]. A biosafe nanofertilizer, a source of P, was found to significantly increase fresh and dry biomass, increase fruit yield, and improve quality by several-times, in addition to leading to a high NUE [96]. Likewise, the application of a nano-sized hydroxyapatite (nHA) in a soybean crop enhanced soybean growth and resulted in a seed yield that was 20.4% higher than that achieved with a synthetic P fertilizer. Similar results were found by Soliman et al. [101], who observed a significant enhancement in the growth and antioxidant contents of *Adansonia digitata* plants treated with the foliar application of nHA. In summary, P applied in the form of NFs can be a suitable option, particularly in smart agriculture, because it has a slow release material over long period, and it can consequently reduce the leaching of P into groundwater and enhance the productivity and quality of crops.

### 5.3. Potassium NFs

Potassium (K) is the third most important macronutrient after N and P, and it has a vital regulatory role in all the physiochemical functions of plants to sustain normal growth and development. Among others processes, K is involved in plant stomatal opening, photosynthesis, the translocation of photosynthates, protein synthesis, ionic balance, water-relationships, and the activation of more than 60 enzymes [17]. Plants with an adequate quantity of K have been shown to be more resistant to abiotic stresses such as water stress and high/low temperatures [50,58,102]. On the other hand, K deficiency negatively affects root shoot growth, the number of seeds inside fruits, size, shape, color, taste, and the final yield of crops [17]. However, the maximum use efficiency of a K fertilizer is typically in the range between 30% and 50% [68], which indicates that up to 50–70% of an applied K fertilizer can be lost, thus causing substantial economic losses and deleterious effects on soil health and water quality [15]. Kubavat et al. [103] studied and developed a nano-potassium fertilizer formulation that had a slow K release rate. The authors concluded that application of a nano-potassium fertilizer could reduce K losses in soil and while sustaining the K supply to crops over a longer period of time. Li et al. [104] observed that K-loaded zeolites increased the yield, harvest index, K concentration, and chlorophyll content in hot pepper (*Capsicum annuum* L.). Similarly, a nano-K fertilizer via foliar application significantly improved the growth, biomass, and quality of *Cucurbita pepo* [105]. Therefore, using K NFs can protect soil health and improve water quality by reducing K losses into soil and leading; subsequently, it can enhance physiological and yield traits.

## 6. Micronutrient (Zn, Fe, Mn, Cu, and Si) NFs and Their Effects on Plants

Though required in considerably smaller amounts than macronutrients, micronutrients are also vital for maximizing plant productivity and quality and for increasing plant tolerance against multiple stresses [8,69,106]. The synthesis of micronutrients by nanosized structures may increase their solubility and bioavailability, aid the obtainment of a more uniform dispersal of these nutrients in the soil, and decrease the adsorption and fixation of micronutrients to soil colloids. 

### 6.1. Zinc NFs

Plant growth significantly depends on Zn nutrition because Zn is a structural component co-factor for various proteins and enzymes. Zinc is also involved in the regulation of auxins, protein metabolism, the biosynthesis of carbohydrates, and the protection of a plant against pathogens and environmental stresses [107]. Zinc NFs in the form of ZnO are frequently used in modern agriculture [21] since they are more efficient and cost-effective than synthetic Zn fertilizers [21,108] and may be used for soil mixing, seed priming [109], and foliar spray [21]. However, trace elements such as Zn can negatively affect plant growth via producing some metabolic alterations in plants if they applied in high doses [110]. Studies have revealed that the application of Zn NFs can increase the germination, seedling growth, yield, and quality of crops [21]. According to Singh et al. [111], ZnO NPs increased the germination of cabbage (*Brassica botrytis* L.) and tomato (*Lycopersicon esculentum* L.), and they improved protein content, sugar content, and antioxidants activities. Likewise, Moghaddasi et al. [112] applied ZnO NPs (100 mg kg^−1^) to cucumber plants and observed that the plants had a higher uptake of ZnO than their synthetic bulk. However, they found that 100 mg kg^−1^ of ZnO NPs inhibited the growth traits of cucumber. Similarly, the application of Z NFs increased shoot growth, leaf area, dry weight, final yield and protein contents in sunflower (*Helianthus annuus* L.), pearl millet (*Pennisetum americanum* L.), rice, maize, sugarcane (*Saccharum officinarum* L.), and potato [21,112,113,114]. In summary, Zn NFs in the form of ZnO are considered the most used NFs in modern agriculture through foliar, soil mixing, and seed priming applications; they are also more cost-effective than synthetic Zn fertilizers. They enhance growth and improve the yield and quality of crops. 

NFs and NPs have been used for improving seed germination and plant growth due their ability to move across seed teguments, where they can increase water and oxygen uptake and can develop resistance against different stresses that affect early plant growth.

### 6.2. Iron NFs

Iron (Fe) is an important nutrient involved in the synthesis of chlorophyll, DNA, chloroplast structure, respiration, and several metabolic pathways. Though plants need the Fe in small quantities for their growth, its insufficiency or excess has detrimental effects on the physiological and metabolic functions of plants, thereby decreasing their yield [115]. Iron availability in well-aerated soils is usually high. However, in these soils, Fe usually forms insoluble ferric compounds at neutral pH values, thus rendering it unavailable to plants. Therefore, Fe-enriched fertilizers could optimize the Fe supply of plants. Different studies have shown that Fe NFs increased germination and improved growth of different crops compared to control and/or synthetic Fe sources. Srivastava et al. [116] documented that iron pyrite NPs increased the growth of spinach (*Spinacia oleracea* L.). Rui et al. [117] observed better root growth in peanut (*Arachis hypogaea* L.) plants treated with Fe NPs compared to non-treated plants under field conditions. Raju et al. [118] observed higher radical length during germination in green gram (*Vigna radiate* L.) and higher fresh biomass with Fe NPs application (2–6 nm) compared to the control (ferrous sulphate; FeSO_4_). Askary et al. [119] used various concentrations (0, 5 10 20, 30, and 40 mM) of Fe NFs (Fe_2_O_3_) on rose periwinkle (*Catharanthus roseus*). They observed that Fe NFs enhanced several growth parameters, chlorophyll and protein contents compared to plants where Fe NFs were not applied. To conclude, Fe NFs can be optimal alternative sources, particularly in soils that suffers from Fe deficiency.

### 6.3. Manganese NFs 

Manganese (Mn) is an essential micronutrient that is involved in N metabolism, photosynthesis and the biosynthesis of fatty acids, ATP, and proteins [115]. In spite of this and depending on the chemical properties of acidic soil, Mn can be toxic to different plants. Manganese also helps plants to cope with different stresses. Research has shown that Mn applications significantly improve the growth and yield of wheat, maize, sugarcane, soybean, and common beans [120,121]. Studies on nano-Mn fertilizers or Mn NPs on different crops have shown that nano-Mn treatments can enhance the root and shoot growth of mung bean by 52% and 38%, respectively, compared to a control treatment (MnSO4; commercially available manganese salt at a recommended dose) [122]. Mn treatments also enhanced the yield of eggplant (*Solanum melongena* L.) by 22% [123] and significantly increased the root length of lettuce (*Lactuca sativa* L.) compared to a control, i.e., Mn ions, as shown in Table 1 [86]. However, there was no effect of Mn NPs on the root length of white mustard (*Sinapis alba*) [124], the seed germination of lettuce [86], or watermelon (*Citrullus lanatus*) yield. At the physiological level, Mn NPs get attached with the chlorophyll binding protein (CP43) of photosystem II, and this results in an increased activity of the electron transport chain and, thus, the overall efficiency of the photosynthesis process [122]. Consequently, plants fertilized with Mn NPs have shown a high rate of nitrogen assimilation and metabolism compared to their conventional bulk counterparts [122].

### 6.4. Copper NFs 

Copper (Cu) is a constituent of regulatory proteins that participates in photosynthesis and respiration of plants and is a cofactor of antioxidants such as superoxide dismutase and ascorbate oxidase. Copper deficiency leads to various disorders; necrosis; stunted growth; low numbers of seeds, grains, and fruits; and finally low crops yield [125]. Soil organic matter content affects the availability of Cu, so the soil application of Cu NPs may be beneficial due to their large surface area, high solubility, and reactivity [81]. In recent studies, the field application of a CuO NPs nanofertilizer improved the germination and root growth of soybeans and chickpeas (*Cicer arietinum* L.) [126]. Likewise, soybean seeds treated with nanocrystalline powders of Cu, Co, and Fr (40–60 nm) had 65%, 80%, and 80% germination rates, respectively, which were higher than the 55% germination rate in a control sample (zero NF) [127]. Similarly, different concentrations of Cu NPs increased the growth and yield of wheat due to improvements in leaf area, chlorophyll contents, number of grains per spike, and grain weight. Moreover, there was an improvement in flavonoid contents, sulphur assimilation, and the biosynthesis of proline and glutathione in *Arabidopsis thaliana* after the application of Cu NPs with dose of 5 mg L^−1^ [73]. Conversely, Cu NP application negatively affected the growth of water lettuce (*Pistia stratiotes* L.) [128] and decreased fruit firmness in cucumber plants [129]. In summary, it seems that Cu NFs can significantly and positively enhance biochemical and yield traits, but one must be careful with their rate application.

### 6.5. Silicon NFs 

Silicon (Si) has been ranked in between essential and nonessential elements for plants because it is not necessary for the completion of a plant’s life cycle. However, it gives certain benefits to some plants under normal and stressful stress conditions [130,131]. Silicon is abundantly present in the earth’s crust; however, soil Si uptake by plants only occurs in the form of mono-silicic acid. Recently, significant attention has been given to Si due to its diverse role in plants against various stresses [131]. For instance, it has been documented that Si can play a substantial role in improving plant tolerance against heavy metal toxicity, as well as heat, water, and salinity stresses [130,131]. Additionally, the application of SiO_2_ with organic fertilizers has the potential to improve overall plant productivity [132,133]. Furthermore, the mesoporous structure of Si NPs enables them to be suitable nanocarriers for various molecules that are beneficial in agricultural systems. For example, nanosensors and nanozeolites, which comprise the structure of Si NPs, are successfully used in agriculture for monitoring soil moisture and enhancing the water retention of soil, respectively [130]. Therefore, Si NPs have the potential to be used as fertilizers for specific plants that cannot survive without a suitable quantity of silicon or as nano-carriers to improve sustainable agriculture.

### 6.6. Born NFs 

Boron (B) is an important micronutrient that has significant roles in elongation of pollen grains and tubes, formation of cellular walls, transfer of photosynthetic organisms from leaves to active sites, and increases in flowers and fruits yields [134]. Studies have shown that B NFs or NPs can improve plant growth and increased yield. Ibrahim et al. sprayed90–180 mg/L of B NPs on mung bean crops, and they reported higher number of pods per plant and a greater seed yield compared to a control (B metal) [135]. Likewise, Genaidy et al. [136] sprayed nano-boron at 20 ppm and nano-zinc at 200 ppm on olive trees, and the plants yielded a maximum number fruits with a high seed oil content. Similarly, Davarpanah et al. [134] reported a greater number of fruits and a higher yield in in pomegranate (*Punica granatum*) after the application of B nanofertilizer (34 mg B per tree^−1^). Taherian et al. [137] applied a B nanofertilizer to an alfalfa (*Medicago sativa*) crop grown on calcareous soil. They harvested a maximum yield with suitable forage quality. In conclusion, B applications of NFs or/and NPs can improve the quality and yield of crops.

## 7. Effects of Physical and Chemical Properties of Soil on NPs

Soil physicochemical properties such as texture, structure, clay minerals, pH, cation exchange capacity (CEC), soil organic matter, and microbial community significantly affect the dispersion, aggregation, stability, immobilization, bioavailability, and transport of NPs [138]. Dissolved organic matter affects the aggregation, mobility, stability, and binding behavior of NPs due to surface charge effects [139]. A higher fraction of exchangeable Ag and ZnO NPs has been noted in soil with a low organic matter content. Soil pH has considerable impact on the bioavailability of NPs. Josko et al. [140] reported that the concentrations of the bioavailable fractions of ZnO NPs and CuO NPs were inversely correlated with soil pH. Soil texture and CEC also affect the movement and adsorption of NPs. Mahdi et al. [141] reported that soil texture greatly affected the transport of Ag NPs, as sandy soils showed a faster transport pattern than fine textured soil. Soils with a high CEC and a high organic matter content were found to have a high adsorption of Ag NPs [142]. Fine texture soils have a high surface area that increases the attachment of NPs to soil particles. Greater attachments of NPs to soil particles leads that NPs with a limited mobility [140]. NPs significantly influence soil microbial activity. CuO NPs and TiO_2_ NPs were found to decrease soil microbial activity and biomass in flooded rice fields [143]. Likewise, You et al. [144] reported that ZnO and Fe_3_O_4_ NPs changed soil microbial communities, decreased biological nitrogen fixation, and influenced soil enzyme activities.

## 8. NFs for Abiotic and Biotic Stress Tolerance 

Abiotic and biotic stresses are major limitations to crop production that have negative impacts on both plant growth and productivity, and they are a main threat to global food security [133,145,146]. Among abiotic stresses, drought, flooding, heat, hail, salinity, heavy metal, and mineral deficiencies are considered to be the main stresses that affect the growth, yield, and quality of crops [8,21,145,146]. On the other hand, different types of insect pests and diseases are biotic stresses that also decrease plant yield. According to the Food and Agriculture Organization (FAO), the main challenge for agricultural scientists is to increase crop production by 70% by the year 2050 [147]. Therefore, the clear identification and appropriate use of novel technologies or approaches to overcome the current yield limiting factors and to increase resource use efficiency are important. At present, numerous studies have shown that the use of NFs or NPs can effectively decrease the adverse effects resulting from different environmental stresses by increasing the levels of plant antioxidant compounds [21,148], (Table 2).

### 8.1. Drought Stress

Drought is a major abiotic stress that significantly decreases agricultural production. Nowadays, the ever-increasing water scarcity problem is negatively affecting agricultural productivity and decreasing the green belts around the world [21]. In addition to the cultivation of drought-resilient crops, the use of stress-ameliorative materials such as NFs has the significant potential to decrease the negative effects of drought stress on plants [149] by increasing the water-holding capacity of soils. Additionally, under stress conditions, the increased production of reactive oxygen species (ROS) cause lipid peroxidation, damages cell membranes, and leads to the leakage of solutes from cells and the death of cells. Studies have shown that NPs can increase the contents of antioxidants and proline, thus decreasing the production of H_2_O_2_ and malondialdehyde [132]. 

Products like Si NPs have been shown to positively increase the tolerance of hawthorn (*Crataegus* sp.) seedlings against drought stress by maintaining the plant’s chemical and physiological functions under stressed conditions [150]. Likewise, Sedghi et al. observed better germination rates (Table 2) of soybean under water stress condition after the application of ZnO NPs (0.5 and 1.0 g/L) compared to a control treatment (0 g ZNO NPs/L) [151]. The foliar spray-application of Fe NPs was found to mitigate water stress effects and to increase yield (Table 2) and oil percentages in safflower (*Carthamus tinctorius* L) [152]. In addition, the foliar spray of 0.02% TiO_2_ NPs was found to increase the tiller number, grain weight, final grain yield, and harvest index of a wheat crop subjected to water stress [153]. Silver NPs ameliorated drought stress effects, improved lentil (*Lens culinaris* Medik) seed germination, and enhanced the dry weight of roots [154]. Finally, the use of Fe NPs with salicylic acid has been shown to increase the drought tolerance of strawberry (*Fragaria × ananassa*) plants [155]. Astaneh et al. [156] reported that the application of nano-chelated nitrogen fertilizer at 41 kg ha^−1^ increased wheat yield under drought stress. Similarly, Mahmoud et al. [157] reported that the application of nano-NPK and nano-zeolite-loaded N reduced the water stress effects and increased the growth of sage (*Salvia officinalis*).

### 8.2. Salinity Stress

The excessive accumulation of Na^+^, Cl^−^, and SO_4_^2−^ ions in the root zone of plants reduces osmotic potential, decreases water uptake, and inhibits plant growth, thus causing the death of plants in some cases [158]. Salt stress is a major issue in dry areas of the world, and more than 20% of globally cultivated lands are affected by salt stress. Excessive salts have negative effects on physiological and biochemical processes such as the photosynthesis, lipid metabolism, protein synthesis, and growth of plants [145]. Salt-affected soils have a low soil osmotic potential, which creates nutritional imbalance in plants and increases specific ionic toxicity. In this context, the use of NFs could be a positive approach to overcome the increasing problems of soil salinity. The application of SiO_2_ NPs was found to increase leaf dry weigh and chlorophyll, proline, and antioxidant contents under salinity stress [159]. Savvas et al. [160] reported that SiO_2_ NPs decreased the Na^+^ ion toxicity and increased the growth of plants under salinity stress compared to plants where SiO_2_ NPs were not applied. Likewise, maize plants grown under salt stress produced higher biological yields with the application of SiO_2_ NPs than plants without SiO_2_ NPs [161]. Similarly, Tantawy et al. [162] used nano-calcium on *Solanum lycopersicum* grown under salt stress, and they reported that plants fertilized with nano-calcium exhibited more fruits per plant and had higher yields (76%) than those grown with synthetic monophosphate. El-Hefnawy [163] applied nano-NPK (50–100 ppm) via foliar spray to pea plants under salinity stress; the nano-NPK alleviated the drastic effects of salinity and increased growth and productivity. Likewise, Zayed et al. [164] reported that nano-chitosan significantly increased the seed germination and growth of bean plant s(*Phaseolus vulgaris* L.) under salt stress.

### 8.3. Temperature and Heat Stress

Extreme temperatures cause oxidative stress and adversely affect the net photosynthesis rates, chlorophyll contents, and growth of plants [165]. Heat stress increases the over-production of ROS, which are, in turn, the main cause of oxidative stress that ultimately results in damage to the lipids in plant membranes and the leakage of ions and solutes However, low doses of Se NPs were found to significantly decrease heat stress effects by improving the water relationships, chlorophyll contents, and antioxidants activities of plants [166]. Under high temperature or heat stress conditions, plants synthesize heat shock proteins that can ameliorate the effects of heat or temperature stresses [162,167]. Studies have shown that multiwalled carbon nanotubes can help plant gene expression for heat shock proteins. When wheat plants subjected to heat stress conditions (air temperatures between 35 and 40 °C) were treated with the foliar spray of Ag NPs (50–75 mg L^−1^) in the trifoliate phase, they showed an increase in growth compared to plants grown with a control treatment (zero AgNPs); the plants treated with 50 and 75 mg/L of Ag NPs showed better root lengths by 5.0% and 5.4%, shoot lengths by 22.2% and 26.1%, root numbers by 6.6% and 7.5%, fresh weights by 1.3% and 2.0%, and dry weights by 0.36% and 0.60%, respectively [168]. The foliar application of 10 mg L^−1^ of Se NPs under high temperature stress during the booting stage of sorghum (*Sorghum bicolor* L. Moench) increased pollen germination, enhanced the system of antioxidant defenses, and thus increased the seed yield of plants compared to those obtained from a control treatment (0 mg L^−1^) [169].

In summary, the application NF or/and NP to plants grown under abiotic stress results an increase in the contents of antioxidants and proline, consequently decreasing the production of H_2_O_2_ and malondialdehyde. Such enhancements can improve the yield and quality of crops. 

### 8.4. Biotic Stress

At a global scale, the annual yield losses because of diseases and pests infestation are estimated to be between 20% and 40% [177]. To reduce the detrimental impact of pests on overall plant productivity, farmers around the world apply millions of metric tons of pesticides every year, which increases environmental pollution, ecosystem disruption, residual toxicity in food and feed, declines in soil fertility, and resistance of insect pests [178]. Different studies have shown that the application of NPs or NFs has the potential to decimate the population of different noxious soil and plant microorganisms, as they can easily enter and disrupt bacterial or fungal cells [179]. Nano-Cu was found to effectively control bacterial diseases (*Xanthomonas campestris* pv. *phaseoli*) in mung crops and the bacterial blight of rice (*Xanthomonas oryzae* pv. *oryzae*) [180]. In another study, Tripathi et al. [181] reported that Cu-Zn bimetallic NPs were effective against yeast (*Saccharomyces cerevisiae*). Chitosan NPs can control fungal, bacterial, and even viral diseases because chitosan NPs bind to microbial cell walls, disrupt cells, alter membrane stability, or attach to DNA and stop replication. Saharan et al. [182] used chitosan-Cu and chitosan-saponin with doses ranging between 0.001 and 0.1%. The authors found that the inhibitory effects of chitosan at a 0.1% concentration decreased the growth of the fungus species *Rhizoctonia solani* (34%), *Alternaria alternata* (82%), and *Macrophomina phaseolina* (87%). Similarly, MgO NPs were found to reduce the growth of *Ralstonia solanacearum* [183]. Cu-based NPs could also be used to kill fungi and bacterial species that affect agricultural plants, as concluded by Ramyadevi et al. [184], who reported of the antimicrobial potential of Cu NPs against various fungal (*Aspergillus niger*, *Aspergillus flavus*, and *Candida albicans*) and bacterial species (*Escherichia coli*, *Pseudomonas aeruginosa, Staphylococcus aureus*, *Klebsiella pneumoniae*, and *Micrococcus luteus*). Nawaz et al. [185] reported that the application of ZnO NPs was effective against the following bacterial species: *E. coli*, *Clostridium perfringens,* and *Bacillus subtilis*. In conclusion, the application of NPs or/and NFs onto infected plants has a high potential to reduce the populations of different noxious soil and plant microorganisms because they can easily enter and disrupt bacterial or fungal cells.

## 9. Effects of NFs on Seed Germination and Growth of Plants

NFs and NPs have been used to improve seed germination and plant growth due their ability to move across seed teguments where they can increase water and oxygen uptake, as well as develop resistance against different stresses that affect early plant growth [185,186]. However, high intercellular concentrations of NFs may stop the seed germination process, reversing the previously mentioned positive effects [25]. As compared to bulk zinc sulphate, the application of nano-ZnO was found to maximize the germination rate of peanut seeds. Likewise, nano-SiO_2_ was found to increase germination in soybean seeds compared to those grown with a non-nano SiO_2_ fertilizer [187]. In addition to their fertilizer effects, NFs can increase net photosynthesis rates by improving chlorophyll contents at the cellular level [25] and reduce the adverse effects of the biotic and abiotic factors faced by seeds during germination [72,84]. In lettuce, for example, germination rates were found to increase following the application of Zn, Cu, Mn, and Fe oxide NPs (<50 mg/L) [188]. Likewise, Ngo et al. [127] documented a significant improvement, compared to a control treatment, in the seed germination of soybeans under field conditions after treating the seeds with Fe, Co, and Cu NPs. The nano-priming (seed priming with NPs) of rice seeds with Ag NPs extracted from *Citrus hystrix* DC (kaffir lime) showed high α-amylase activity, germination rates, and numbers of healthy seedlings [189]. However, seed coating with NFs or NPs does not guarantee a normal or enhanced germination rate in all cases, and other environmental conditions such as proper soil moisture and temperature have been shown to be more determinant for seed germination [190].

## 10. Effects of NFs on Yield and Quality of Plants

Several field and greenhouse studies have reported yield benefits following the application of different NFs and NPs. The foliar application of NPK NFs was found to enhance the yield and yield parameters of chickpeas [191]. Tarafdar et al. [113] reported that zinc nano-fertilizer increased the grain yield of pearl millet (*Pennisetum americanum* L.) by 37.7%. The foliar application of ZnO, MgO, and CuO NPs increased cotton yield to between 18% and 23% [192]. Singh et al. [25] observed a higher achene yield of sunflowers fertilized with ZnO than those with other treatments.

The use of NFs can also increase the quality of agricultural products. For instance, Afshar et al. [193] reported higher Zn and protein contents in seeds without yield penalties following the application of a Zn NF. In cowpeas (*Vigna unguiculata*), the application of nano-Fe increased the seed protein content by 2% compared to Fe from synthetic fertilizers [175]. In forage maize, Sharifi et al. [113] found that Zn and Fe NFs applied via foliar application progressively enhanced the crude protein, P, and carbohydrate contents, as well as biological yield, when compared to plants grown with synthetic fertilizers. In sunflowers, Sham [194] observed that ZnO NPs applied as a foliar spray increased both the achene carbohydrate and oil contents when compared to other treatments.

## 11. NFs for Developing Smart Agriculture

In the coming decades, the agriculture sector will face increasing pressure to provide food security for a rapidly increasing world population without increasing its overall environmental footprint. One option to attain higher biomass and grain yields could be the modification of present fertilization techniques. Nutrients such as N, P, K, Ca, Mg, Cu, and Zn are crucial for the growth and reproduction of plants, and these nutrients are provided to plants in the form of synthetic fertilizers that have experienced a continuous increase in the rate of adoption among farmers around the world since the green revolution [195]. Though the yield of crops has remarkably increased since the use of chemical fertilizers in the early 1960s, the NUE values of these fertilizers are low, which commonly results in the application of supra-optimum rates to achieve higher yields and, consequently, greater nutrient losses to the environment. Therefore, sustainable efforts are being made to synchronize nutrient availability and improve NUE values in agricultural systems without a further deterioration of surrounding environments [196]. The use of smart fertilizers like NFs has been proposed as a way to increase the overall NUE values of fertilizers through a more controlled, and slower nutrient release that could better match the sustained nutrient needs of crops across time [24,197]. The consistent and slow release of nutrients for extended periods of time can be achieved by using semipermeable coatings (which control the solubility of the fertilizers in water or soil solutions) on the surfaces of or within fertilizers [198]. This will lead to a new framework of fertilizers that will deliver an accurate amount of nutrients at the right time, as well as a dramatic reduction of nutrient losses to the environment. Furthermore, nanosensors can be attached with NFs or NPs to deliver specific nutrients to targeted sites within living systems.

Nanosensors are a promising tool and have significant potential in agriculture and food production. Nanosensors are extremely tiny devices or nanodevices that can be attached to whatever is wanted to be detected. These tiny sensors detect and respond to physical, chemical, and biological process, and they transfer the responses into signals that can be processed by humans [199]. Nanosensors provide real time information about field conditions, crop growth, pesticides, and plant diseases, and they can help predict environmental stressors [200]. Yao et al. [201] used fluorescent nanoprobes of silica NPs for the detection of bacterial spot disease in *Solanaceous* plants caused by *Xanthomonas axonopodis*. Similarly, Sharon and Sharon [202] synthesized carbon nanomaterial-based chemical sensors for the detection of pesticide residues in plants. A nano-sensing system turns conventional agriculture into a precision farming system, and real-time monitoring has reduced the over-application of fertilizers and pesticides, which is helpful in protecting the environment from contamination [203]. Prasad et al. [204] reported that nanobiosensors can monitor glyphosate and glufosinate herbicides in soil using nanofilm-modified pencil graphite electrode.

## 12. Factors Affecting the Uptake and Translocation of NPs by Plants

As seen in the above-mentioned sections, the taking up and translocation of NPs by plants are influenced by some factors such as the NPs themselves (e.g., size and surface functionalization), plant morphology and physiology (e.g., root integrity, leaf shape, age of the plant, exposure conditions, plant species, and stomatal dimensions), and interactions of NPs, the environment, and rhizosphere processes. The size of NPs is one of the main limitations for diffusion into plant cells, and previous investigations have reported maximum NP absorption and accumulation limits for plant cells (i.e., up to 50 nm) [29,50,51,58], although some other investigations have stated different diameters of NPs (i.e., >50 nm) [61,64,65]. Furthermore, NP type and chemical composition can prompt the taking up and translocation of NPs into plant cells [71,82,84,93,150,151,171,172]. Additionally, the coating surface of NPs can significantly affect the characteristics for their uptake and accumulation via plant cells [24,40,190,198].

Pérez-de-Luque [205] reviewed and summarized the factors that can influence the absorption, uptake, transport, and penetration of NPs in plants as follows: (A) the application method of NPs, whether it foliar or soil application; (B) in soil application, NPs can interact with some microorganisms that have positive symbiotic relationships with plants such as fungi (e.g., mycorrhiza) and bacteria (e.g., rhizobacteria), as well as some compounds (e.g., humic acids and organic matters) that can ease their bioavailability in rhizospheres and their absorption and taking up by plants. Meanwhile, salt ions can induce and obstruct the uptake of NPs. Mainly, NPs have to pass and cross through different tissues (e.g., epidermis and endodermis) and barriers (e.g., cortex, Casparian strip, and cuticle) to get into the vascular tissues (e.g., phloem, companion cells, and xylem) subject to the access pathway (e.g., root or leaf); (C) NPs can move up and down a plant through the apoplastic or/and symplastic pathways; (D) NPs can enter plant cells through numerous mechanisms such as pore formation, endocytosis, plasmodesmata, and carrier proteins. 

In exogenous application, NPs must translocate through the barrier of the cuticle, subsequently crossing the lipophilic or hydrophilic pathways [206]. The lipophilic pathway comprises diffusion via cuticular waxes, while the hydrophilic pathway is achieved via polar aqueous pores existing in the stomata or/and cuticle because the diameter of cuticular pores is about 2 nm [54]. The morphological dimensions of stomata are about 25 μm of length and 3–10 μm of width [54]. Nevertheless, as a result of the physiological function and unique geometric structure of stomata, the real size exclusion limit of stomatal holes for NP diffusion is still unclear. This means that the pathway through stomata seems to be the most likely pathway for NP diffusion because it can allow NPs with a maximum diameter 10 nm to pass [54]. In exogenous application, NPs are preferred to be applied with a low exposure dosage at different stages, suitable weather conditions, and higher leaf area indexes to avoid nutrients loss [207].

## 13. Limitation of Using NFs in Terms of Ethical and Safety Issues

Though NF and NP technologies have the clear potential to revolutionize the agriculture sector and its productivity, some of these advantages may come at a high cost. There are various safety and ethical issues pertaining to the application of NFs or NPs in agriculture systems, as exposure to NPs and NFs could result in health risks. In this situation, unintentional health safety and environmental issues can limit the application of NPs or NFs in agricultural crop production [7]. Furthermore, NPs and NFs can enter the food chain, thereby increasing their dissemination in non-targeted living organisms. Though direct disease transmission from the use of NFs or NPs to human beings has not been reported at present, some studies have shown that NPs or NFs could induce adverse biological responses [208]. As such, there is a scientific need to understand if NFs are completely changed into ionic forms and assimilated into different metabolites and proteins once taken up by plants or if some residues remain intact and must later be moved into other non-targeted living organisms in the food chain. Some studies have shown that NPs can alter gene expression in animals because of their size, which allows them to enter different animal tissues, cells, and organelles and then interact with DNA [209]. NFs like macronutrient and micronutrient fertilizers are currently being used in agriculture [16], but the use of supra-optimum application rates may lead to the deposition of nano-based macro and micronutrients and cause nanotoxicity and a reduction of water quality [16].

NPs affect living organisms in different ways, e.g., carbon-based NPs modify DNA structure and the expression levels of genes in plant tissue [210,211]. ZnO NPs affect symbiotic relationships in legumes and delay the nitrogen fixation process [212]. They also cause nutritional imbalance and induce molecular changes in plants, e.g., CuO NPs have been found to affect hormone (e.g., indole-3-acetic acid and abscisic acid) levels in plants [213]. Iron-based nanomaterials (nFeOx) affect the hydraulic conductivity of roots due to particle aggregation on the root surface, which results in a low uptake of water and nutrients such as Ca, K, Mg, and S [214]. 

## 14. Conclusions and Future Prospects

From the sustainable agriculture perspective, nanotechnology has the potential to develop new innovative types of fertilizers such as NFs to increase global food production to feed the increasing world population. NFs have potential as part of smart crop production systems under the framework of sustainable agriculture because they have large surface areas and a characteristic slow and steady release of nutrients. These promising characteristics make them highly suitable for use in modern agriculture.

The use of NFs can increase agricultural productivity and resistance against biotic and abiotic stresses. Therefore, the use of NFs in the agriculture sector cannot be ignored. The application of NFs may help to decrease the amount of fertilizers via the smart delivery of active ingredients, to increase nutrient uptake and NUE values, and to decrease fertilizer losses from volatilization, leaching, runoff, and consumed energy during production. Furthermore, the use of seed coatings with NFs and nanosensors may decrease the costs of agricultural production and environmental issues.

NFs can release their nutrients in 40–50 days, while synthetic fertilizers do the same in 4–10 days. As a result, synthetic fertilizers, particularly N-urea, can rapidly lose more than 50% of nutrient contents after field application through leaching and volatilization. However, research has shown that NFs release nutrients as much as 12 times slower than synthetic fertilizers, and they can significantly increase the yields and quality traits of crops. The foliar application of NFs is much better and preferred than the soil application of NFs due to its significant enhancements in the growth, physiological and biochemical traits, yield, and quality of crops—particularly in smart agriculture.

The research-based and judicious use of NFs must be studied in detail before the marketing or distribution of NFs at the commercial scale. Future studies must be focused on the safety, bioavailability, and toxicity of different NFs or NPs used for agricultural production. Furthermore, bio-synthesized or green synthesized nano-biofertilizers and NFs should be explored in order to further increase yields in sustainable agriculture.

## Figures and Tables

**Table 1:** The effects of different NF and/or NP types and their dose ranges on different crops.

NFs/NPs	Range of Doses	Plant/Crop	Effects	Reference
Zn NFs	5–20 mg/L	*Allium cepa* L.	Reduced root growth	[71]
Zn NFs	100–500 ppm	*Capsicum annuum* L.	Increased seed germination	[72]
Zn NFs	500 mg/kg	*Pisum sativum* L.	Reduced H_2_O_2_ and chlorophyll molecules	[73]
Zn NFs	1000 mg/kg	*Cucumis sativus* L.	Inhibited root growth	[74]
ZnO NPs	20 mg/L	*Triticum aestivum* L.	Increased biological and grain yield	[75]
ZnO NPs	10 mg/L	*Cyamopsis Tetragonoloba* (L.) Taub.	Increased growth, biological yield, and nutrient contents	[76]
ZnO NPs	10 mg/L	*Zea mays* L.	Increased root shoot length, plant height, leaf area, chlorophyll content, and grain quality	[77]
ZnO NPs	5–20 mg/L	*Solanum melongena* L.	Reduced germination, root length, and leaf area under culture media but increased these parameters under soil conditions	[78]
Cu NPs	20–80 mg/kg	*Coriandrum sativum* L.	Decreased germination and shoot growth	[79]
Cu NPs	50–500 mg/L	*Solanum lycopersicum* L.	Increased antioxidant contents and fruit firmness	[71]
Cu NPs	10–20 mg/L	*Lactuca sativa* L.	Decreased seedling growth and dry weight of seedlings; affected water relationships and nutrient contents	[80]
Cu NPs	130–660 mg/kg	*Lactuca sativa* L.	Increased shoot/root length ratio	[81]
CuO NPs	500 mg/kg	*Triticum aestivum* L.	Increased biological yield	[82]
Cu NPs	200 mg/kg	*Spinacia oleracea* L.	Increased fresh biomass and photosynthesis rate	[83]
Fe-based NFs	30–60 ppm	*Pisum sativum* L.	Increased chlorophyll contents and seed weight	[84]
Fe-based NFs	10–20 mg/L	*Lactuca sativa* L.	Increased antioxidants and enzymatic activities but decreased overall growth	[80]
Nano-iron oxide (Fe)	500–1000 mg/L	*Cuminum cyminum* L.	Increased stem length, yield (130%), and Fe concentration in plant (110%)	[85]
FeO	1–50 ppm	*Lactuca sativa* L.	Germination was maximum at 1 ppm of FeO but a high root length was noted at 10 ppm	[86]
FeS_2_	80–100 µg/mL	*Cicer arietinum* L.	High germination rate and crop yield	[87]
Nano-nitrogen (N)	25–100%	*Oryza sativa* L.	Increased tillers per plant, height, and dry weight	[88]
Nano-apatite (P)	100 mg/L	Soybean	Increased biological yield (18.2%) and root length	[89]
Hydroxyapatite (P)	200 mg P/kg	*Lactuca sativa* L.	Increased P content and dry weight	[90]
Nano-potash (K)	1500–2500 mg/L	*Arachis hypogaea* L.	Increased shoot length, stem diameter, biological yield, and number of flowers per plant	[91]
Chitosan-NPK (500, 60, and 400 ppm; respectively)	10%, 25%, and 100%	*Triticum aestivum* L.	Increased P and K contents but decreased protein content	[92]
MgO	7–10 µg/mL	*Solanum lycopersicum* L.	Decreased bacterial wilt disease caused by *Ralstonia solanacearum* L.	[93]
MnO	0.25–50 ppm	*Lactuca sativa* L.	No effect on germination but increased root length	[86]
Nano-silica (SiO_2_)	30–60 mg/L	*Triticum aestivum* L.	Increased relative water content (84%) and final yield (18–25%)	[94]
SiO_2_ NPs	15 kg/ha	*Zea mays* L.	Improved growth parameters	[95]
Sulfur NPs	500–4000 ppm	*Vigna radiata* L.	Increased dry weight	[96]

**Table 2:** The effects of different methods application of NF and/or NP types on different crops grown under different environmental stresses.

NFs/NPs	Method of Application	Stress	Plant/Crop	Effects	Reference
Fe	Foliar	Drought	*Carthamus tinctorius* L.	Reduced effect drought and increased yield	[152]
Fe		Drought	*Fragaria ananassa*	Increased drought resistance in the field	[155]
ZnO		Drought	*Glycine max* L.	Increased germination	[151]
SiO_2_		Drought	*Crataegus* sp.	Increased photosynthesis by improving stomatal conductance and increased yield	[150]
Na_2_SeO_4_		Heat	*Lycopersicon Esculentum* L.	Improved water relationships of plants and increased chlorophyll contents	[166]
Se		Heat	*Lycopersicon Esculentum* L.	Increased growth and yield	[169]
CuO 20–2000 μg/mL	Pre-sowing	Oxidative stress	*Allium cepa* L.	Increased antioxidant activities	[71]
SiO_2_ 1.5–7.5 g/L	Pre-sowing	Salinity	*Cucurbita pepo*	Increased germination, photosynthesis, and antioxidants; decreased production of H_2_O_2_	[170]
Nano-urea Hydroxyapatite 25–100%	Pre-sowing	Salinity	*Prunus dulcis* L.	Increased germination plant height, and secondary roots/plants, yield	[171]
Chitosan-Cu 10 mg	Post-transplanting	Salinity	*Solanum lycopersicum* L.	Increased plant growth and gene expression for jasmonic acid	[172]
Si 1–5 mg/L	Post-transplanting	Salinity	*Capsicum frutescens*	Increased salt tolerance	[72]
Nano-Ca 0.5–1 g/L	Post-transplanting	Salinity	*Solanum lycopersicum* L.	Increased flowers/plants, yield and improved stem diameter	[173]
Nano-silicon 1–2 mM	Foliar application	Salinity	*Jatropha integerrima*	Enhanced vegetative parameters and chemical constituents	[174]
Na_2_SiO_3_ 10 μM P	Post-transplanting	Heavy metal	*Pisum sativum* L.	Decreased uptake of heavy metal and increased antioxidants activities	[175]
SiO_2_		Salinity	*Ocimum basilicum*	Increased fresh and dry weights and chlorophyll and proline contents	[158]
SiO_2_		Salinity	*Glycine max* L.	Increased antioxidant enzymes and decreased oxidative stress	[148]
ZnO		Salinity	*Helianthus annuus* L.	Increased CO_2_ assimilation and photosynthesis rate; reduced Na content in leaves	[176]
SiO_2_		Mineral nutrient	*Carthamus tinctorius*	Increased yield	[132]
Zn		Mineral nutrient	*Pennisetum americanum*	Improved leaf area, chlorophyll content, and enzyme activities	[113]
